# Image Segmentation Using Encoder-Decoder with Deformable Convolutions

**DOI:** 10.3390/s21051570

**Published:** 2021-02-24

**Authors:** Andreea Gurita, Irina Georgiana Mocanu

**Affiliations:** Computer Science Department, University Politehnica of Bucharest, RO-060042 Bucharest, Romania; andreea.gurita@upb.ro

**Keywords:** image segmentation, convolutional neural network, Xception model, deformable convolutions, mean intersection over union

## Abstract

Image segmentation is an essential step in image analysis that brings meaning to the pixels in the image. Nevertheless, it is also a difficult task due to the lack of a general suited approach to this problem and the use of real-life pictures that can suffer from noise or object obstruction. This paper proposes an architecture for semantic segmentation using a convolutional neural network based on the Xception model, which was previously used for classification. Different experiments were made in order to find the best performances of the model (e.g., different resolution and depth of the network and data augmentation techniques were applied). Additionally, the network was improved by adding a deformable convolution module. The proposed architecture obtained a 76.8 mean IoU on the Pascal VOC 2012 dataset and 58.1 on the Cityscapes dataset. It outperforms SegNet and U-Net networks, both networks having considerably more parameters and also a higher inference time.

## 1. Introduction

Computer vision is an interdisciplinary field. It seeks to automate the tasks involved in human vision by helping computers to get a better understanding of what is depicted in images or videos.

Image segmentation is part of the computer vision field and is the process of partitioning an image into distinct regions. Each region has pixels that have similar characteristics. More precisely, this task assigns a label to every pixel in an image so that the pixels with the same label share similar characteristics and properties such as color, texture.

The goal of segmentation is to turn the image into something meaningful and easier to analyze it, by working with objects from the image instead of pixels.

A practical application of image segmentation is represented by content-based image retrieval (finding similar images in large databases) [1]. Additionally, image segmentation has many applications in the medical field, where it can be used for different purposes, such as: locating tumors [2], surgery planning, virtual surgery simulation, diagnosis, and many others. Another useful application of image segmentation consists in object detection or pedestrian detection, face detection and recognition, and fingerprint recognition. Image segmentation can also be used for traffic control systems, video surveillance, and autonomous driving.

Initial segmentation techniques group pixels by exploiting accessible information such as close spatial locations and global attributes (e.g., color); the newer approaches also take into account also the context of the pixels.

Multiple algorithms and techniques that are analyzed in different studies have been developed for image segmentation [3,4,5]. General approaches that are primarily known in this area are (1) based on thresholding (pixels are allocated to classes based on the range of values in which it lies), (2) edge-based methods (an edge filter is applied to the image, (3) pixels are classified by the position according to the edges (using the Sobel or Laplacian operator), (4) region-based methods (neighboring pixels with similar values are grouped, if different values there are not in the same group), (5) segmentation based on clustering (a cluster refers to a collection of similar elements), etc. The modern techniques are supervised learning methods based on convolutional neural networks (CNNs).

Initially, CNNs were used for classification, where usually, the network output is a single class. By analyzing the majority of the existing networks developed for image segmentation, it can be easily observed that most of them are adaptations of CNNs that were designed for image classification.

The paper proposes a network for image segmentation using a convolutional neural network that achieves competitive mean intersection over union (IoU) compared with some of the existing methods evaluated on public datasets. The scope of the proposed network is to obtain a mean IoU comparable with existing other networks while having a low number of parameters and somewhat short inference time.

The main contributions of this paper are as follows:

- we propose a network for image segmentation that combines parts of the existing networks—it uses an encoder-decoder architecture based on the Xception classification model, that maintains a significant reduction in the number of parameters;
- the proposed network does not use only the traditional layers as it makes use of a new and improved architecture by adding a deformable convolutions module. Additionally, it takes advantage of residual links that bring improvement over the classic serial networks;
- results show that the proposed architecture achieves comparable results with other existing networks (that have similar number of parameters).

The paper is organized as follows: Section 2 describes the main related work models, showing relevant information regarding the architectures along with their performance on the original ones, description of three public datasets (Pascal VOC 2012, Cityscapes and ADE20K), metrics that are used for performance evaluation and evaluation of the related work models tested on these three datasets. Section 3 presents the proposed architecture along with all the experiments that were made in order to improve the performance of the proposed architecture using the evaluation on the three datasets. Section 4 contains the analysis of the performance of the proposed method and comparison results with existing methods. Conclusions and future work are given in Section 5.

## 2. Related Work

This section presents some existing models that are used for image segmentation together with their own original performances. In order to propose a new method for image segmentation, described methods were evaluated on three public datasets: Pascal VOC 2012 [6], Cityscapes [7] and ADE20K [8].

### 2.1. Existing Solutions for Image Segmentation

A modern approach for image segmentation models consists in an encoder-decoder structure [9], where the input first is downsampled. In the second part, the feature maps are upsampled to produce a full-resolution segmentation map. The downsampling maps have a lower-resolution, but they are efficient at discriminating between object classes, as they are more specialized.

Another type of approach is based on Fully Convolutional Networks (FCNs), meaning that it only has locally connected layers such as convolution, pooling, and upsampling layers, but no dense layers. FCN has a few advantages as it has small number of parameters and computation time. Another advantage consists in working with original image size. One such example is given in [10], who built a FCN-U-Net that achieves better segmentation for medical images. The proposed architecture, consists of two paths: a contracting path and a symmetric expansion path. The contracting path is built by the consecutive two 3 × 3 convolutional layers, each layer followed by a rectified linear unit (ReLU) and 2 × 2 max-pooling layer. Its purpose is to capture the context. The second path, also known as the expanding path, is built by the consecutive application of 2 × 2 up-convolutional layers of the feature maps, which is followed by the concatenation with the corresponding crop feature map from the first path and two 3 × 3 convolutional layers, each followed by a ReLU unit. This path enables precise localization by allowing the network to propagate context information to higher resolution layers. The network uses an overlap-tile strategy that enables the use of images of different sizes as inputs. The proposed architecture was tested on a few datasets. The first evaluation was made on segmentation of neuronal structures applied in electron microscopic recordings. The result is measured in “warping error” (0.000353), the “Rand error” (0.0382), and the “pixel error” (0.0611). Another dataset tested was for cell segmentation in light microscopic images. On the first data set “PhC-U373”, IoU was 92%, while on the second data set “DIC-HeLa”, the mean IoU was 77.5%. The advantages of the U-Net network consists in reaching a high accuracy given an adequate dataset, and training time. It uses a fully convolutional network, and it does not depend on the input size. As a disadvantage, the size of the U-net must be comparable with the size of features. Because it uses many layers, the training time can be high.

Another encoder-decoder structure was introduced in [11]. They introduced an architecture called SegNet based on a deep FCN that has as purpose pixel-wise segmentation. It is similar with U-Net network—it consists of an encoding part and a decoding part. The encoding part is similar to the convolutional layers from the VGG16 network designed for object classification. Therefore, it is easier to initialize the training process from weights trained for classification on large datasets. By discarding the fully connected layers, the number of parameters of the SegNet is reduced compared to other architectures. The decoding part maps the low-resolution feature maps from the encoder network to higher input resolution feature maps using pooling indices computed in the max-pooling step. The upsampled maps are then convolved with trainable filters to produce dense feature maps. These are followed by a pixel-wise classification layer that provides class probabilities for each pixel. The encoding network is built by convolution layers that produce a set of feature maps that are then batch normalized and after that a rectified linear unit is applied, followed by a 2 × 2 max-pooling layer. The architecture was tested on the CamVid dataset, where it got a higher performance compared to other models: mean IoU per class of 71.20, and mean IoU of 60.10. It was also tested on a large dataset, SUN RGB-D, where it reached a mean IoU of 31.84 and mean IoU per class of 44.76. Additionally, SegNet is efficient both in terms of memory and computational time during inference and has a small number of parameters.

Paper [12] proposed a new convolutional network that uses dilated convolutions (also known as atrous convolutions) to combine contextual information at multiple scales without losing resolution. Atrous convolutions support the expansion of the receptive field without loss of resolution or increasing the number of parameters. The network is based on the VGG architecture [13] (which is a pretrained network previously used for classification). They added a module, called front-end module, that can be subsequently used in any existing architecture. From VGG, they removed the last two pooling layers. The next convolutional layers are replaced with atrous convolutions. The proposed module has seven layers that apply a 3 × 3 convolution with different factors of dilation. These layers are followed by one layer that applies a 1 × 1 convolution and produces the output. The module reaches mean IoU of 69.8 on the Pascal VOC 2012 validation set, and mean IoU of 71.3 on the test dataset. Using only the frontend it reaches mean IoU of 71.3, with frontend and context the result is 73.5, with frontend, context, and Conditional Random Field it reaches 74.7. By using all this combined with a recurrent neural network architecture the mean IoU is 75.3. Results proved that the frontend module improves accuracy in all the performed experiments. A disadvantage of atrous convolutions is that they are computationally expensive. They also use a significant amount of memory having to be applied to a large number of high-resolution feature maps.

Another model for image segmentation—DeepLabv3—is proposed in [14]. The architecture is also based on a previously used architecture for image classification, ImageNet [15], which they pretrained in order to get better and faster training. The main focus of the paper was to remodel the DeepLab architecture. It outperforms its predecessors, even with the post-processing step removed. The network is based on ResNet blocks [16]. They also employ the use of atrous convolutions, for increasing the field of view without requiring learning extra parameters. A dilated convolution operator is a convolution operator that is modified to use the filter parameters differently. The dilated convolution operator can apply the same filter at different ranges using different dilation factors. Besides the use of atrous convolution with various rates, they also experimented with other different methods, including the use of atrous spatial pyramid pooling (ASPP) (laying out the dilated convolutions in parallel). As experiments, they first tried to analyze the impact of the training protocol, by changing the learning rate policy, the crop size, adding batch nominalization, and data augmentation. They also experimented with atrous convolutions to build more blocks in cascade and with ASPP. They reached the mean IoU of 81.3 on Cityscapes and 86.9 on Pascal VOC 2012.

A continuation of this work is DeepLabv3+ [17], as they try to use the advantages of the encoder-decoder networks as U-net and SegNet, the ASPP module (they encode multi-scale contextual information with the help of multiple fields-of-view). The network reaches the mean IoU of 89.0 on Pascal VOC 2012 and 82.1 on the Cityscapes datasets. The deep convolutional neural network used as an encoder is based on the Xception network [18]. They replaced the max-pooling layer with depthwise separable convolutions and added extra batch normalization. After the encoder, they placed the ASPP. The decoder is a simple network that also takes advantage of skip connections in order to retrieve some of the information from the beginning of the input image.

### 2.2. Datasets

In order to develop a new model for image segmentation, based on existing solutions, we analyzed the performances of the main segmentation models on three public datasets: Pascal VOC 2012, Cityscapes and ADE20K (existing methods were tested on other datasets than the ones they are already tested).

PASCAL VOC 2012 Dataset [6] has its main goal to recognize objects from several visual object classes in realistic scenes. The dataset is composed of twenty object classes (Table 1) and around 11,000 images.

The training dataset consists of a set of images; each image has an annotation file giving a bounding box and object class label for each object in one of the twenty classes present in the images. Multiple objects from multiple categories may appear in the same image. The data has been split into 50% for training/validation and 50% for testing. The distributions of images and objects by class are approximately equal across the training/validation and test sets.

The Cityscapes Dataset [7] contains images of urban street scenes. There are three types of annotations: semantic, instance-wise, and dense pixel annotations. There are 30 classes of objects taken from 50 cities during different months (in spring, summer, and fall), taken during the daytime. There are a total of 5000 annotated images with fine annotations and 20,000 annotated images with coarse annotations. The classes are shown in Table 2.

ADE20K dataset [8] is one of the most extensive open-source datasets for semantic segmentation and scene parsing, released by the MIT computer vision team. In the dataset, there are 20,210 images in the training set, 2000 images in the validation set, and 3000 images in the testing set. Each image has an annotation file providing the label of the object for each object in one of the 150 types present in the image. Multiple objects from multiple categories may be present in the same image (an image contains at least five objects, while it can also go up to over 250). This shows the high annotation complexity of the ADE20K dataset. There are eight object classes frequently annotated with their corresponding parts: person, building, car, chair, table, sofa, bed, lamp in order to do some part-segmentation.

### 2.3. Metrics for Evaluation Image Segmentation

The Intersection-Over-Union (IoU) [19], also known as the Jaccard Index, is one of the most commonly used metrics in semantic segmentation. The IoU is the area of overlap between the predicted segmentation and the ground truth divided by the area of union between the predicted segmentation and the ground truth.

For binary or multi-class segmentation, the mean *IoU* of the image is computing by averaging the *IoU* of each class. Additionally, IoU can be computed based on *TP* (number of true positive pixels), *FP* (number of false positive pixels), *TN* (number of true negative pixels) and *FN* (number of false negative pixels) as given in the following formula:

$$IoU=\frac{TP}{TP+FP+FN}$$

**Pixel accuracy** [19] represents an alternative metric to evaluate semantic segmentation by reporting the percent of pixels in the image that were correctly classified. It can be computed as in the following formula:

$$Pixel\_accuracy=\frac{TP+TN}{TP+TN+FP+FN}$$

### 2.4. Evaluation of the Existing Methods

Models described in Section 2.1 applied to image segmentation: U-Net [10], SegNet [11], DilatedNet [12] and DeepLabv3 [14]) were evaluated on three public datasets: Pascal VOC 2012, Cityscapes and ADE20K.

We trained the four networks for the PASCAL VOC 2012 dataset (size of the input images was 160 × 160). Results for the other two datasets (Cityscapes and ADE20K) were extracted from the existing benchmarks. Evaluation was performed based on the mean IoU. Results are given in Table 3.

As we can see, the best mean IoU is reached by DeepLabv3. The second mean IoU value is obtained by the DilatedNet.

For the Pascal VOC 2012 dataset, both DeepLabv3 and DilatedNet have greater training time than the U-Net and SegNet (the training time for DeepLabv3 is 25 h 7 m, for DilatedNet is 23 h 19 m, for SegNet is 6 h 40 m and for the U-Net is 3 h 35 m on a local computer). Additionally, the number of parameters are different, such as: U-Net and SegNet that have similar number of parameters (30 million parameters) and DeepLabv3 and DilatedNet which have almost double the number of parameters).

The main benefit of DeepLabv3 is that it provides the best performance among similar solutions in multiple benchmarks and can easily be adjusted to more complex tasks. Its downside is the higher training time, which can be remediated by using pre-training weights.

ADE20K is one of the more complex datasets, and due to the high number of classes and the complexity of the images, the networks tend to score poorly compared to the previous two datasets.

Although the U-Net network does not have the best performances, a significant advantage is the lowest training time. It also does not rely on the input size. A disadvantage of this network is that in order to have good results, the size of the network has to be comparable with the size of features. As a result, the training time is most probably to increase.

Thus, we used as a starting point an architecture similar to either U-Net or SegNet, using an encoder-decoder architecture. Both U-net and SegNet are based on conversions of convolutional networks that had initially been designed for image classification—in this case VGG16.

VGG16 [13] is a convolutional neural network model that chieves 92.7% top-5 test accuracy in ImageNet [20] (14 million images, 1000 classes). It replaces large kernel-sized filters (11 and 5) with multiple 3 × 3 kernel-sized filters one after another. While VGG achieves high accuracy, the training is challenging on modest GPUs because of substantial requirements for computation, both in terms of memory and time.

As an improvement, in [21] was introduced the Inception micro-architecture that behaves as a multi-level feature extractor. It computes 1 × 1, 3 × 3, and 5 × 5 convolutions within the same module of the network. The outputs of the filters are stacked along the channel dimension and before being fed into the next layer in the network. It approximates a sparse CNN with conventional dense construction.

ResNet network introduced in [16] allows the training of deep networks by constructing on top of micro-architectures called residual models. ResNet achieves better accuracy than VGGNet and Inception while being computationally more efficient than VGGNet.

A new and better architecture called Xception was proposed by [18] and outperforms Inception, VGG16, and ResNet. The architecture is based on a couple of architectures (VGG-16 and Inception) and combines different ideas such as residual connections and depthwise separable convolution layers. A depthwise separable convolution [10] works with kernels that cannot be turned into two smaller kernels, like in the VGG architecture. This type of convolution deals not just with the spatial dimensions, but with the depth dimension (the number of channels) as well. The Xception architecture [18] outperforms also Inception on the ImageNet dataset—the architecture has the same number of parameters as Inception, but the performance gains are due to the more efficient use of model parameters.

Therefore, in order to maintain both training time and number of parameters at lower values, we started with an encoder-decoder architecture that is based on the classification model—Xception architecture.

## 3. Proposed Solution

Based on the architectures analyzed in Section 2 for image segmentation, we decided not to use an architecture similar to DeepLabv3 or the DilationNet because, even though they have quite good performance in terms of segmentation results, both training time and number of parameters are too high compared with U-Net [10] and SegNet [11] architectures.

We tried to achieve suitable performance without compromising the training time and spatial complexity—to create a simple network in terms of depth—that employ the use of a large number of high-resolution feature maps.

Thus, we proposed an architecture based on an encoder-decoder network architecture (similar with the U-Net and SegNet architectures), that does not only use the traditional layers, but also improved architecture and types of layers, such as depthwise separable convolution. In addition, it takes advantage of residual links that can bring improvement over the classical serial networks. Since both U-net and SegNet are based on conversions of convolutional networks that had initially been designed for image classification we started with an architecture based on an encoder-decoder architecture that is based on classification models. As shown in Section 2.1 we chose as the classification model, the Xception architecture (using on its currently performances).

The base architecture is an adaptation of the Xception network to the task of semantic segmentation. Next we made different optimizations in terms of training and architecture modifications. The following aspects were evaluated in order to see if the performances of the network can be increased:

1. using transfer learning;
2. increasing the input resolution size;
3. increasing the depth of the network by adding modules to the middle flow;
4. adding data augmentation;
5. using optimizers.

We started from the base architecture and applied the previous steps. At each step we made experiments with different values. For each step, the network with the best performances was chosen to be extended in the next step. After that, the best obtained model was fine-tuned and the following experiments were performed on it:

1. using dilated convolutions;
2. using atrous spatial pyramid pooling;
3. using deformable convolutions.

Thus, the final version of the network is an encoder-decoder architecture based on the Xception network adapted to the segmentation task, fine-tuned (using the Adam optimizer, freezing the first two modules), and adding a deformable convolutions module (based on the architecture proposed in [22]) along with a skip connection.

The base architecture is described in Section 3.1 and each improvement of the initial network is detailed in Section 3.2.

### 3.1. Base Architecture

**Figure 1:**
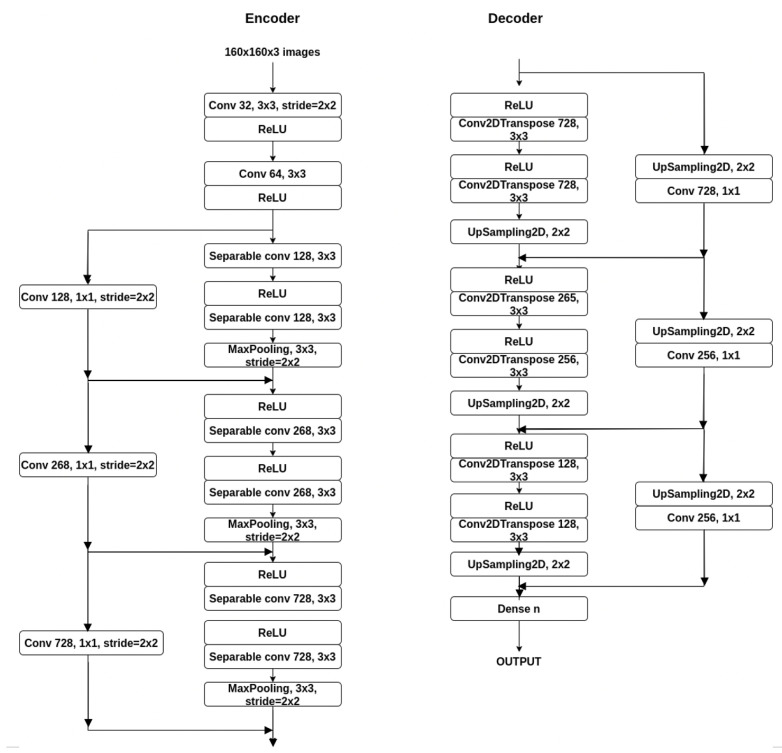
The proposed architecture, the decoding branch is almost symmetric to the encoding branch, except it reconstructs the values to the input image size using deconvolutions and upsampling layers.

The base architecture is an encoder-decoder architecture (Figure 1).

Since the Xception architecture currently has the best performances (its accuracy is higher than the Inception, ResNet and VGG16), we started with a base architecture that uses parts from Xception network that are adapted for the semantic segmentation task. Thus, we took the entry flow and turned it into the encoder part.

The results of the network applied on the three datasets are given in Table 4.

In the preprocessing part, the input image was scaled to a smaller value (in order to be able to train the network faster, the value was 160 × 160). The annotated image, called the target image was also scaled to this value but turned to grayscale.

The encoder part is built using Modified Depthwise Separable Convolution (MDSC) [23]. The architecture is relatively similar to the one used in Xception, except that we removed the second convolutional layer and we have only two layers surrounded by residual links. At the output of the encoder part, the size of the input image was shirked to a quarter, so we need the decoder part to up-sample the image, but also learn segmentation features. For the decoder part, the opposite of the ending part it is usually used, meaning mostly the inverse layers. For max-pooling, as it minimizes the size of the input, we need to up-sample with the same value.

To the encoder part, we added a symmetric decoder, which up-sampled the images. The output of the network is in the size of the input image in height and width, and the number of classes in depth. For max-pooling, as it minimizes the size of the input, we need to up-sample with the same value. For the convolutions, in the case of the separable ones, we need to use a transpose matrix (sometimes called deconvolution). The first modules of the decoder part are the opposite of the ones that are used in the encoding branch, having the same values.

Overall, the model has very low performance in terms of the mean IoU, but not very bad in terms of pixel accuracy. The model performs poorly due to the lack of enough data, necessary to train the model on a significant number of classes (especially for ADE20K dataset, where are 150 classes). The difference between the accuracy and mean IoU values is because the classes that appear more often are made of multiple pixels and are easier to be recognized than smaller objects.

The inference speed of the proposed model is less than 2 s, which is not too much, but very high in terms of real-time performance. The inference time was computed on an Intel Core i5, while the training was done on Google Colab, Tesla K80, with 2496 CUDA cores, 12GB GDDR5 VRAM.

### 3.2. Architecture Optimization

The architecture performs well in terms of speed of training and spatial complexity, but due to the depth, which is not very high, it has problems in recognizing multiple classes.

The network performs poorly on these three datasets because it is not deep enough to learn all the features as other existing networks. The advantages are in terms of speed of training and spatial complexity.

**Figure 2:**
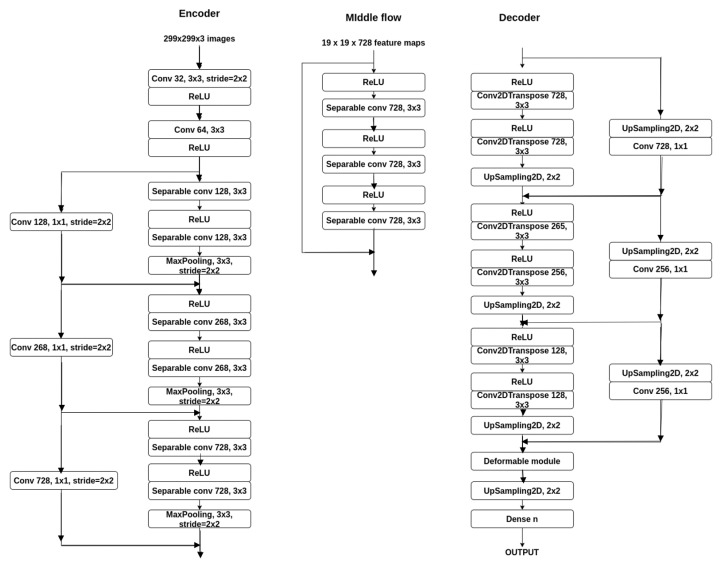
The proposed architecture, with resolution 299 × 299 and different number of modules for the middle flow (between 2 and 8).

**Figure 3:**
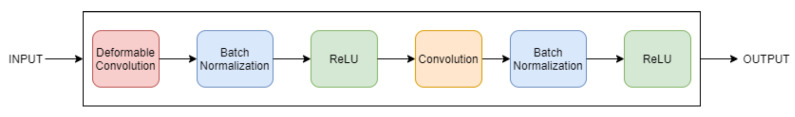
Proposed module based on ASPP and Adaptive Context Encoding (ACE).

**Figure 4:**
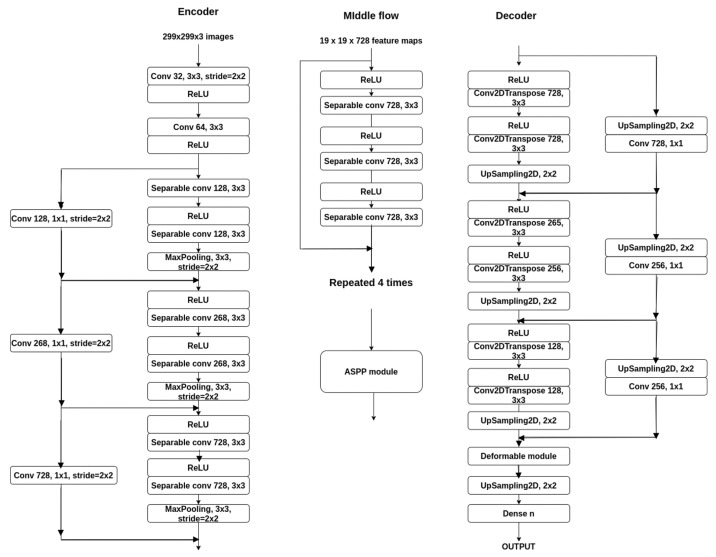
Architecture of the best proposed model.

Thus, we made the following experiments in order to check if the performance of the network can be increased without substantially increasing the number of parameters of the network:

1. Using transfer learning: In many cases, there is not enough labeled data available to train a model. Thus, a model that was previously trained on one task is reused in another task. In case of image segmentation, many people use a model trained on ImageNet. In our case, we want to improve the performance while taking advantage of the small and fast network. Thus, we used the Xception weights after it was trained on the ImageNet dataset (Xception achieves 79% Top-1 accuracy and 95% Top-5 accuracy on this dataset).
  Using the same architecture as stated before, but only adding pre-train on the down-sampling branch, we obtain the results given in Table 5 tested on the three datasets. As we can see, transfer learning brings some improvement in terms of pixel accuracy and mean IoU, but still, the performance is not yet comparable with the models from the state of the art.
2. Increasing the input resolution size: We have to consider the fact that the network resolution may be too small to predict classes very accurately. Therefore, as a next step, we increased the input resolution size to the default size of the Xception network (299 × 299), almost doubling the size. We chose this value because the network was trained on original images, and it reached the highest performance.
  The new network now has 14 million parameters (also added a Crop2D layer that helps us deal with the extra pixels that can occur because the odd size when down-sampling had a different value when up-sampling). Adding multiple layers brings the drawback of increasing the training time.
  The network with the increased input resolution size was tested both with and without transfer learning. The results obtained by this method can be seen in Table 6.
  The performance was increased, even without transfer learning. Increasing the resolution brings a performance gain for all cases. Transfer learning also helps this new network, but the mean IoU is still very low compared to the other existing methods.
3. Adding modules from the middle flow to increase the depth of the network: We experimented with the Xception architecture again, by adding different numbers of modules from the middle flow, in order to see who much increasing the depth of the network would affect the performance.
  An Xception module from the middle flow is built of hree separable convolutions, each with 728 filters of size 3 × 3, along with their corresponding ReLU activation function. In Figure 2, the new architecture is presented.
  We tested the architecture after adding two modules, four modules, six modules, or eight modules (the entire middle flow). First, we tested this part only on the Pascal VOC 2012 dataset in order to obtain the variant with better results). For this part, we computed the pixel accuracy and the mean IoU for both resolutions: 299 × 299 and 160 × 160 and along with transfer learning (as it proved to be the most helpful so far).
  The results can be analyzed in Table 7 (for resolution 299 × 299) and Table 8 (for resolution 160 × 160).
  As we can see, too many layers can make the model overfit, such as when using eight additional modules. The model learned the data very well on the training set. The results are better for resolution 299 × 299, thus the number of parameters were computed only for this resolution.
  Using the best model obtained for Pascal Voc 2012 dataset (299 × 299 resolution and the extra four modules), the result on the Cityscapes dataset are: 88% pixel accuracy and 47 mean IoU, and on ADE20K dataset, 71% pixel accuracy and 27 mean IoU.
4. Adding data augmentation: Some of the datasets that we used have too little data in order to have a meaningful training. Data augmentation encompasses techniques that enhance the size and quality of training datasets such that we can build a better model by incorporating them [24]. Also, too much augmentation may pull down the performance.
  We added the following transformations for the dataset:
  - Random contrast change for the input image
  - Random hue change for the input image
  - Random brightness change for the input image
  - Random scale for the input image and annotated image (same value)
  - Random crop to fit input size (along with mean padding when necessary on the image, 0 padding when annotated image)
  - Random flip left-right of the input image and annotated image (same flip)
  We used the transformations along with a data generator for the dataset. Therefore the same image can appear in the dataset multiple times with different transformations. We trained the model for the same number of epochs, with the best model that we obtained until this step: resolution 299 x 299, with transfer learning and four extra modules from the middle flow, and the performance was increased. Table 9, shows the obtained results on the three datasets.
5. Using optimizers: we experimented few changes to the architecture regarding the optimizer used and also different techniques for fine-tuning were applied (as a part of the network is from Xception, which was also pretrained). We trained the following optimizers (based on the performances described in [25]) using the previous models:
  - Stochastic gradient descent (SGD): some advantages of the SGD compared with momentum [26] are that the model usually converges faster. Still, as a disadvantage, it has a high variance in model parameters, and it may overshoot even after achieving global minima.
  - Adagrad [27]. As advantages, it eliminates the need to tune the learning rate manually, learning rate changes for each training parameter, and it is capable of training on sparse data. Still, it is also computationally expensive and slow. It also suffers from decaying learning rates.
  - Adadelta [28] is an extension of Adagrad, which tries to remove the decaying learning rate problem. As a disadvantage, it is computationally expensive. Another advantage is that there is no need to set the default learning rate (the running average of the previous time steps is taken).
  - RMSprop [29] tries to resolve the same problem. Additionally, the learning rate gets adjusted automatically, and it chooses a different learning rate for each parameter.
  - Adam [30]. The method is fast and converges rapidly, computationally efficient, and has minimal memory requirement.
  - The Rectified Adam optimizer [31]: compared to Adam, it can obtain higher accuracy and complete training in fewer epochs. They found that adaptive learning rate optimizers (such as Adam) struggle to generalize during the first few batch updates and have very high variance. They solved the problem by applying warm up with a low initial earning rate and turning off the momentum term for the first few sets of input training batches.
  The results obtained by testing these optimizers with the proposed model are given in Table 10. (each model was run three times, and the average is shown). From these results, we can see that some optimizers perform better than the others, Adadelta being the one that performed the worst on all datasets. As a surprise, Rectified Adam could not outperform Adam nor SDG (on Pascal VOC 2012, ADE20K), but this is mostly because of the matter it has to solve. Adagrad also performed very well on all datasets (achieving the highest accuracy on Pascal VOC 2012), while others only managed to accomplish performance in one of them (SDG showing the high variance it suffers from).
  RMSprop performed reasonably well on all, only not the best. From now on, we used Adam optimizer, as it achieved the best results in terms of mean IoU on two of the three datasets, and second-best on the other.
  Next, we made fine-tuning of the network in order to improve its metrics. From the previous experiments, transfer learning works very well and brings high gains (using pre-trained weights of the Xception network). Next, we made some improvements regarding the learning rate and freezing the first few layers, that are frequently used techniques for fine-tuning. Fine tuning of the parameters was performed for each dataset. First, the beginning few layers’ weights were frozen, as they capture universal features like curves and edges, and we may not want to change them. By doing this, we get the network to focus on learning dataset-specific features in the subsequent layers. We decided not to try every one of the possible layers but only every two. We initially only froze the first layer of the network (Conv2D), then the third, the fifth, and so on. The results are given in Table 11. The best performance on all datasets was when the first two modules were frozen in the beginning, thus they only learn high-level features. As we started freezing more layers, the performance tended to decrease—the network had a harder learning time.
  After seeing that freezing the first five-six layers brings the best performance, we tested how the learning rate increases impacts the network. In this step, the results do not include also freezing the layers.
  In previous experiments, we used the Adam optimizer, which also proved to be the best for our problem. The entry and middle flow of the network are similar to the ones in Xception (except the fact that the intermediate flow of the proposed model has only four out of the original eight modules), and that network was also trained with the same optimizer. The initial learning rate stated [1] is 0.045, after which they use a decreasing learning rate, with a decay of rate 0.94 every two epochs. In the previous steps, we tested the network with the default learning rate (which is 0.001).
  Next, we used the approach introduced by Smith [32], an automatic learning rate finder algorithm. In this case, we need a range of values to test for, in our case, it is between 0.0001 and 1. The network is then trained with a learning rate for a batch, starting with the smallest one, and after the batch, the rate is exponentially increased. For the proposed model, on both datasets (Pascal VOC 2012 and Cityscapes), the best learning rate that we can use is the range [0.0002, 0.0006].
  In contrast to these two datasets, on ADE20K, the best learning rate is between [0.001, 0.01] because of the complexity of the dataset with the high number of classes, which makes the loss much larger. The leaning rate does not affect the performance between a broader range too much, but it could help if it is chosen close to the values with the smallest loss. If we choose a lower rate than the minimum in the interval, the network fails to learn the particular features for the specific datasets, and the loss is very high. If we choose a bigger learning rate, the network overshoots; therefore having a hard time finding the local optima.
  The highest metric values are reached for the rate of 0.0005. For Pascal VOC 2012 pixel accuracy is 91.93% and the mean IoU 61.43, while for Cityscapes pixel accuracy is 87.41% and mean IoU 45.73, and 0.001 for the ADE20K dataset: pixel accuracy is 75.7% and mean IoU of 28.29%.
  By combining the two types of fine-tuning: freezing the first six layers of the network, (fine tuning of the parameters was performed for each dataset) along with using the learning rate of 0.0005, we get the following values:
  - for Pascal VOC 2012 the pixel accuracy is 90.18% (increasing 4.48%), mean IoU is 60.72 (increasing 1.36%);
  - for Cityscapes the pixel accuracy is 88.37% (increasing 0.07%) and mean IoU is 47.86 (increasing 2.74%);
  - for ADE20K the pixel accuracy is 73.41% (increasing 2.11%) and mean IoU 27.24 (increasing 0.06%).
6. Using dilated convolutions: As simple convolutions might struggle to integrate global context, dilated convolutions [33] helps to increase the field-of-view without increasing the computational cost. We started adding the dilation rate parameter of the separable convolutions in the original network parts of the upsampling modules. The upsampling branch does not get changed, for the obvious reason. A significant advantage for these experiments is that the number of parameters stays the same, but the metrics can highly improve. As a first experiment, we changed the entry flow. The entry flow is composed of two regular convolutions (that we froze in the previous tests), along with three modules, each consisting of two separable convolutions (along with batch normalization and activation) and a max-pooling layer. In parallel to each module, there is a skip connection from the entry of the module.
  From the entry flow, we tried three experiments:
  - in each module to make the first separable convolution to be atrous
  - in each module to make the second separable convolution to be atrous
  - in each module to make both separable convolutions to be atrous
  Now the next problem we encountered is what dilated rates we should choose for these convolutions. Based on the existing research we used an increasing dilation rate, starting from 2 and then doubling it with every layer [10, 12, 32].
  Therefore, we experimented with the following values: convolutions from the same module have the same dilation rate, while the rates increase with the power of 2 representing the order in which they are: so for the first module we use the dilation rate 2, for the second module we used dilation rate 4 and for the third module, the dilation rate 8.
  The result of the experiments is presented in Table 12. The best result on all datasets is when only the first convolution from all three modules is dilated. When dilating all convolutions, we can see that the model loses the accurate localization of small objects, therefore decreasing the metric values.
  For the second experiment, we changed the middle flow of the network. The middle flow is composed of four modules. This time, a module is a little more complex compared to the module from the entry flow. Each module is composed of three separable convolutions (along with batch normalization and activation). In parallel to each module, there is a skip connection from the entry of the module. From the middle flow, we tried four experiments:
  - in each module to make the first separable convolution to be atrous
  - in each module to make the second separable convolution to be atrous
  - in each module to make the third separable convolution to be atrous
  - in each module to make all three separable convolutions to be atrous
  Similar to the previous experiment, convolutions from the same module have the same dilation rate, while the rates increase exponentially by 2 representing the order in which they are: so for the first module we use the dilation rate 2, for the second module we used dilation rate 4 and for the third module, the dilation rate 8 and for the fourth 16. The result of the experiments are given in Table 13.
  The best result on all datasets is when we add a dilation rate only to the third convolution in the module. In this case, the network even outperforms the previous experiment, when we dilated the convolutions in the entry flow. The worst result when dilating particular layers was when we dilated only the first convolution. This is probably because we only learn small separated parts of the objects and then learn the next layers based on this information. The worst result overall is when using dilated all convolution as we tend to lose the accurate localization of objects.
  We also tried combining the best result from the first experiment with the best result from the second. On the Pascal VOC2012 dataset, we obtained pixel accuracy of 87.91% and mean IoU of 60.54. On the Cityscapes dataset, pixel accuracy was 88.09% and mean IoU 49.02. The pixel accuracy was 62.92% and the average IoU 16.56 on the ADE20K dataset. The results are smaller than the best result when dilating only the third convolution of the modules from the middle flow but a little bit higher than the ones when the first convolution was atrous from the modules of the entry flow. The decrease in performance comes without surprise because, as we previously have seen, too many dilated convolutions decrease the performance.
7. Adding atrous spatial pyramid pooling: Atrous spatial pyramid pooling was first introduced in DeepLabv2 [33] helps to account for different object scales, which can improve the accuracy. The ASPP used is very similar to the one used in DeepLabV3+. The output of the input is passed through a 1 × 1 convolution layer, an image max-pooling layer, and three convolutions with different dilation rates, all in parallel. The result is then concatenated and goes through another 1 × 1 convolution. In experiments, we added the ASPP module after the middle flow, before the decoder.
  For the experiments we conducted, we varied the rates to see how it impacts the network. For this part, we removed the dilated convolutions from the previous experiment as we want to see if using this kind of module outperforms simple atrous convolutions in the network. As a future experiment, we combined both of them. We tested the following rates: (3, 6, 9), (6, 12, 18), (12, 24, 36) and (24, 48, 72). The result can be inspected in Table 14.
  The best results on the first two datasets are obtained by using the (12, 24, 36) dilation rates, this achieves a larger field of view compared to the previous atrous rates, and therefore, it can gather more contextual information, and also, improve the metric values of the network. The values then tend to drop because as the sampling rate becomes larger, the number of valid filter weights becomes smaller, and information can no longer be captured, decreasing the performance metrics. On ADE20K, the best result is for the (3, 6, 9) atrous rate of the ASPP module. This difference comes from the complexity and the high number of objects in one image, as too high of a rate would make the model lose contextual information. Using deformable convolutions: In comparison to dilated convolutions, which have a larger but fixed dilation value during convolution while deformable convolution, different dilation values are applied to each point in the grid during convolution [34]. In paper [22] is proposed a model for context encoding (Adaptive Context Encoding—ACE) based on deformable convolution, which outperforms the atrous spatial pyramid pooling—ASPP modules at the segmentation task on PASCAL and ADE20K. Unfortunately, the authors do not mention exact values used for the block, but they state that the blocks consist of “Deformable Convolution, Batch Normalization, Rectified Linear Unit”. Based on this description, we created a block consisting of a deformable convolution, Batch Normalization, activation, a standard convolution, another Batch Normalization, and activation. The proposed block can be inspected in Figure 3.
  Firstly, we tested the network with a single deformable convolution after the middle flow. Then, the following architectures are with the module specified previously, and a structure of three blocks positioned in parallel (how it is in ASPP) and in cascade. The results are presented in Table 15. The network performs the best when a single module is used after the middle flow of the network.
  Using this kind of module brings the best performance so far, with a total of 26 million parameters. In order to improve it even more, we used the technique of adding a skip connection from the first few layers of the network (Figure 4) so to bring back some information about the object bounds that we may have lost through the multiple convolutions. The obtained results on the evaluated datasets are given in Table 16.

## 4. Discussion

In order to implement the network, we used Python and the open-source neural network library Keras.

First we built an image data generator, which saves memory space while training the model. First, data was loaded from the tensor slices saved in some tfrecord file. Then data was shuffled, in order to have different images for each training step, parse and decode. The next step consists in applying transformation for data augmentation using TensorFlow image functions:

- Random contrast change for the input image
- Random hue change for the input image
- Random brightness change for the input image
- Random scale for the input image and annotated image (same value)
- Random crop to fit input size (along with mean padding when necessary on the image, 0 padding when annotated image)
- Random flip left-right of the input image and annotated image (same flip)

For building the model, we first employ the Xception model that is present in Keras, to which we add the layers we stated. We already previously mentioned the layers used along with the number of filters or stride (when it is the case). We also added batch normalization after almost every convolutional layer, as it may help improve the speed, performance, and stability of the network. Most of the layers used are already in the Keras library, so it was easy to employ their use. An exception are the deformable convolutions. For this type of layer, we used a custom layer based on Conv2D that deals with the offsets. The optimizer used is Adam, with a fixed learning rate, that we previously found. We experimented with the optimizers and learning rates in order to find the one that gets the network to maximal performance.

For the loss function, we use sparse categorical cross-entropy, as the targets are integers representing the classes. Cross entropy measures the dissimilarity between the distribution of observed class labels and the predicted probabilities of class membership. At the same time, categorical refers to the fact that we can have more than two classes. Another loss function we tried is the Jaccard index, that measures similarity between finite sample sets and is defined as the size of the intersection divided by the size of the union of the sample sets.

The model with the best performance is the one we obtained after fine-tuning (using the Adam optimizer, with a learning rate of 0.0005, freezing the first two modules and batch size 48), and adding a deformable convolution module along with a skip connection. First, we analyzed the datasets to see which classes appear more often.

The code was run on a TPU v3-8 (for the best model). We added specific code that initializes it and calls a TPU distribution strategy implementation. In order to visualize the results (after the network was trained and tested), we used the Python Imaging Library. We preprocessed the image as previously stated, using the evaluate function was used. The result of the network is then processed using the argmax to find the most probable class for each pixel and then replace the pixels with the recommended colors from each dataset.

### 4.1. Dataset Analysis

As a first step to determine the performance, we need to analyze the datasets we used. It could help in determining why some classes are easier or harder to segment, as maybe some classes appear more often than others or are built out of more or less pixels.

In the Pascal VOC 2012 dataset, there are 6144 images available to the public, which we split into a train set (3264 images), a validation (1440 images), and a test set (1440 images). As previously stated, there are 21 classes in the dataset, and the images have between two and six categories, the majority being two (66% of them) and the least six (0.1% of the images). In terms of what classes appear in the most images in the dataset, the ‘background class’ appears in all of them. The next category is ‘person’ (25% of images), followed by ‘cat’ (over 9%). The least frequent class is ‘sheep’, appearing in only 4% of the images. For this dataset, the most frequent class in terms of pixel occupation over all the images is the dataset is the ‘background’ class (around 90% of pixels in all images). The second frequent is ‘person’ with almost 2%. The rest of the classes occupy under 1% of all pixels in images.

The Cityscapes dataset has 4669 images available, which we split into a train set (2576 images), a validation (568 photos), and a test set (1525 photos). This dataset has 31 classes, of which only 19 can be found in the public dataset. The images in this dataset have between 8 and 19 classes present in a single image, with the majority being 19 (53% of images) and very few with eight or nine classes. It instantly shows that this dataset has a much higher complexity compared to the previous one. It also has fewer images, so most of the models may have a high difficulty learning a complex segmentation on little data. In this dataset, eight classes appear in all images (‘background’, ’road’, ’sidewalk’, ‘building’, ‘wall’, ‘fence’, ‘pole’ and ‘traffic light’). The next six appear in over 97% of the images (‘traffic sign’, ‘vegetation’, ‘terrain’, ‘sky’, ‘person’, and ‘rider’). The class ‘truck’ appears in 71% of the images, followed by ‘bus’ (66%), ‘train’ (62%), and ‘motorcycle’ (61%). The least frequent class is ‘bicycle’ which appears in only 57% of the images. The most frequent class in terms of pixel occupation over all the images is the dataset is the ‘background’ class (with 44% of the pixels), followed by ‘sidewalk’ (with 20% of the pixels—which is expected as it is a dataset with urban street scenes), ‘traffic sign’ (with 14% of the pixels), ‘rider’ (with 6%) and ‘road’ (with 5% of pixels). The least frequent classes are ‘motorcycle’ (0.08%) and ‘person’(0.015%). The rest of the classes occupy between 3% and 0.2% of images.

In the ADE20K dataset, there are 17136 images available to the public, which we split into a train set (10104 images), a validation (1488 images), and a test set (5544 images). ADE20K is a very complex dataset having 150 classes. The images have between 1 and 148 classes per image, most having between 4 and 11. The first class in this dataset appears in every image, while the first 61 classes appear in over 10% of the images. The minimum appearance for a class is 1%. Additionally, in terms of pixel occupation of images, the background class occupies 56%, while the next has a high drop in number. It is followed by 7%, the first nine have all over 1%, and after class 107, the appearance drops under 0.1.

### 4.2. Model Performance

For the three datasets we analyzed how well every object is segmented.

For the Pascal VOC 2012 dataset, the mean IoU for each class is shown in Table 17. Based on the mean IoU per class, the best and worst cases are the following:

- best cases: ’background’ and ’bus’ classes;
- worst cases: ‘bicycle’ and ‘potted plant’ class (the size and the small number of pixels that represent the class);
- most of the classes are very well recognized (20 out of 21 with over 50% IoU, 15 out of 21 with over 70% IoU).

**Figure 5:**
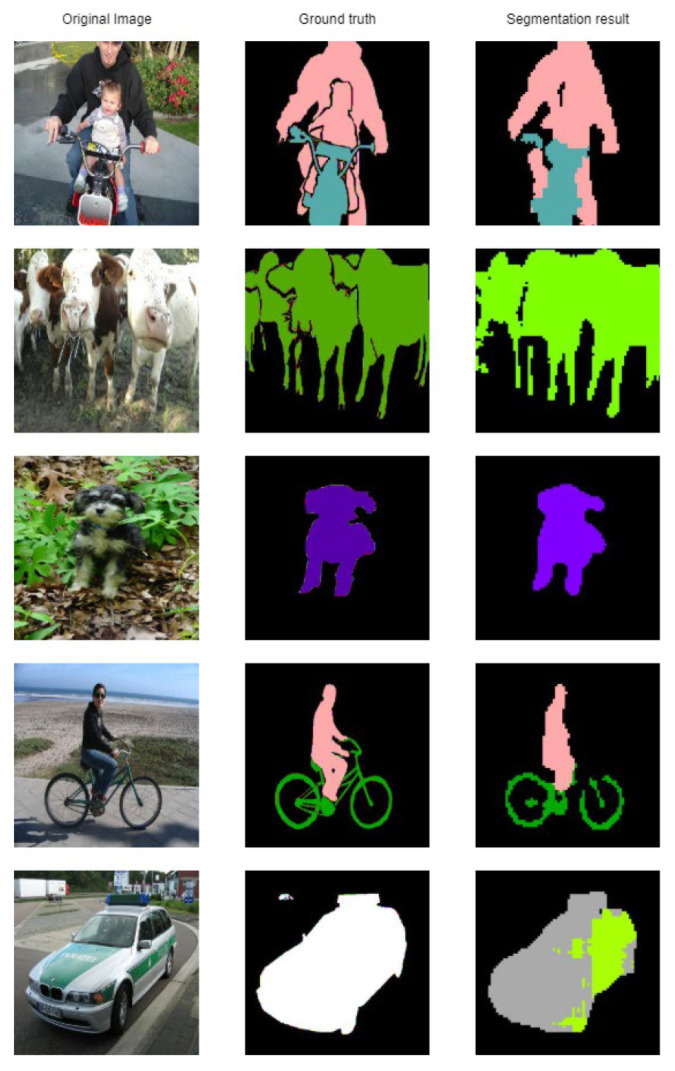
Segmentation result on classes on the Pascal VOC 2012 dataset [6].

Classes as ‘bicycle’ and ‘sofa’ are usually confused with ’background’. Other classes that look similar are sometimes confused, such as: ‘chair’ and ‘sofa’, ‘dog’ and ‘cat’, ‘sheep’ and ‘cow’. Additionally, classes ‘person’ and ‘sofa’ are confused, probably stems from the images where people are seated on a sofa. Some example results are given in Figure 5.

It can be seen that some classes are segmented almost ideally (‘dog’, ‘cow’), while ‘bicycle’ miss a significant number of pixels. Small pixels from the vehicle are confused with a ’cow’, probably the combination between the black window, green and white color of the car.

For the Cityscapes dataset, the mean IoU for each class is shown in Table 17. Based on the mean IoU per class, the best and worst cases are the following:

- best cases: ’background’ and ’sidewalk’ classes;
- worst cases: ‘fence’ and ‘pole’ (they have small number of pixels/class) ‘bus’ and ‘train’ (that have similar looking), ‘sky’ and ‘person’, ‘wall’, and ‘sidewalk’ (that have similar textures), ‘person’ and ‘rider’ class (the difference consists only in the context of being on a bike or not and the network is not able to learn it).

**Figure 6:**
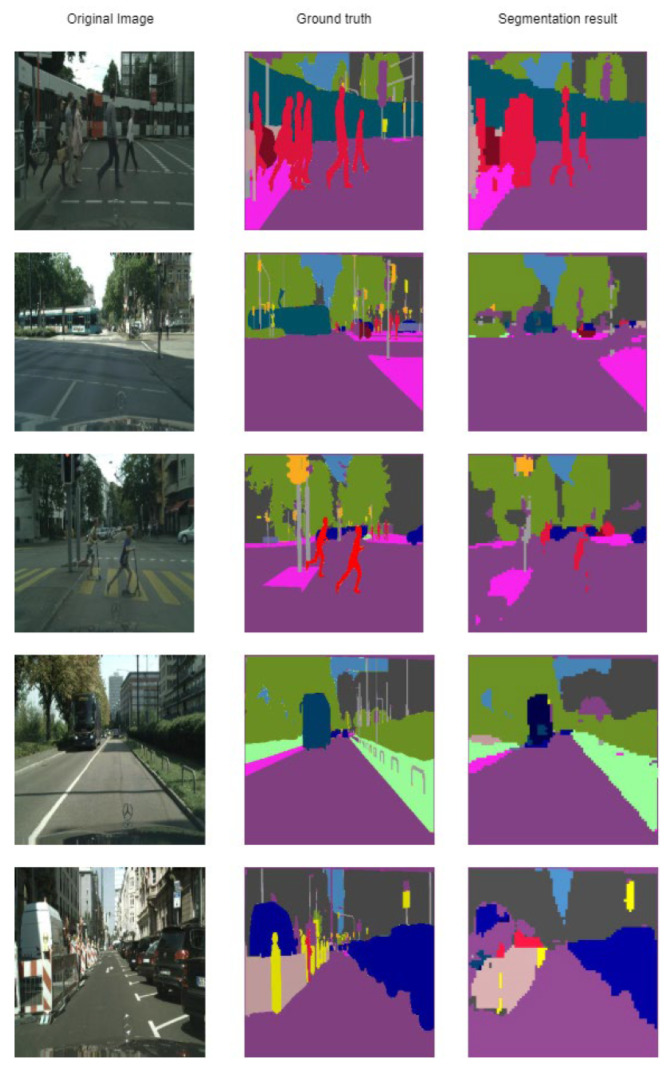
Segmentation result on classes on the Cityscapes dataset [7].

Thus, the objects with the highest ‘recognition’ are those that are very different (for example vegetation – the color green does not appear very frequent in a street landscape), terrain (in this case representing grass or horizontal vegetation) and traffic signs. For this dataset, usually, the more prominent objects are very well segmented. Some obtained results are given in Figure 6. In contrast, the smaller ones (such as pedestrians—which in this case are confused with the sky) are usually missing a lot of pixels.

**Figure 7:**
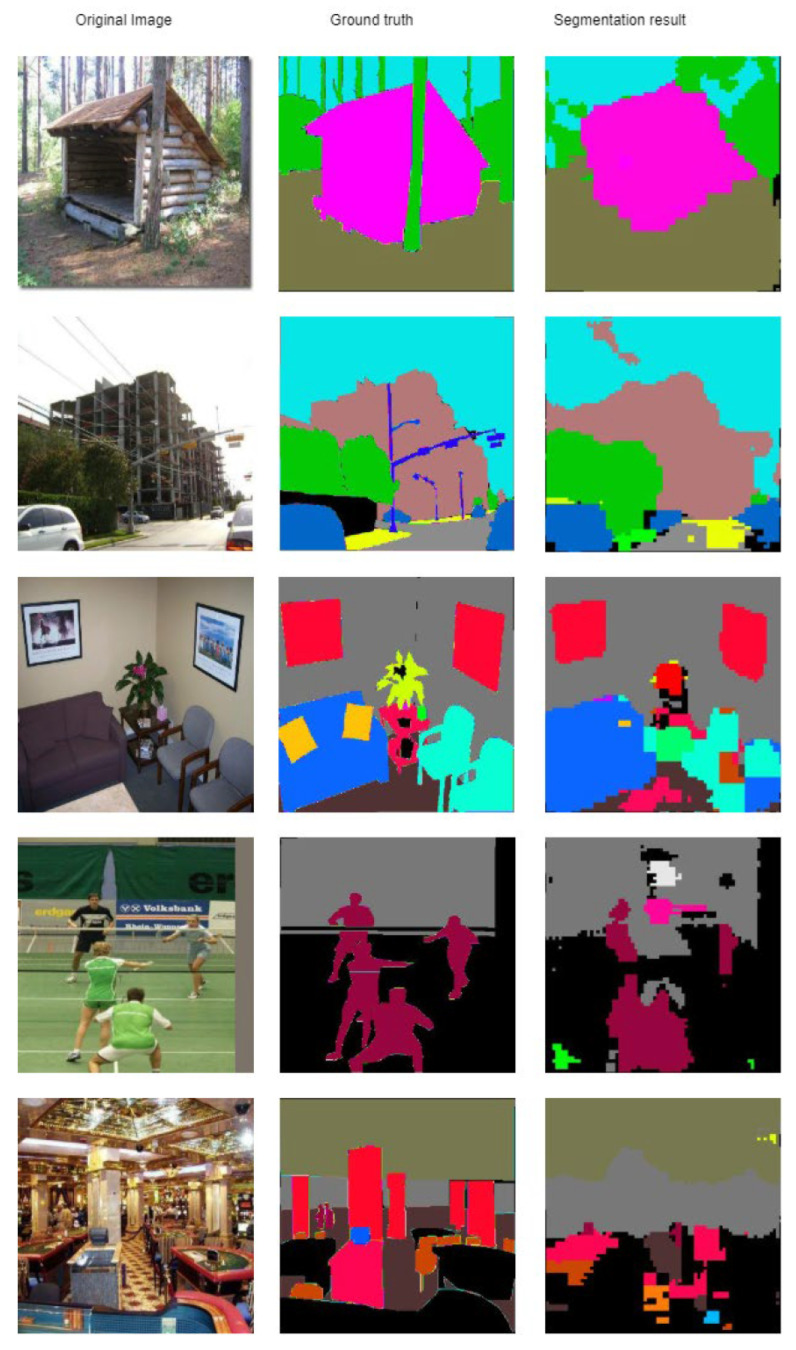
Segmentation result on classes on the ADE20K dataset [8].

Some results obtained on the ADE20K dataset are given in Figure 7. The bigger objects are segmented relatively well. Usually, the problems appear at smaller, often ‘rarer’ objects or the objects that are visually harder to differentiate. As favorable examples we can see the tree that is in front of the wooden house (first line—both objects are made from the same material) and the casino (fifth line—where the columns and ceiling have similar colors).

Based on the obtained results, the proposed model segments better bigger objects. The model tends to miss smaller objects, especially in an image where a significant number of objects are presented (such as most images from the Cityscapes and ADE20K datasets). The model also mixes up similar-looking objects. Multiple instances of the same object that are in proximity also tend not to be very well distinguishable in the resulting segmented image.

### 4.3. Performance Against Related Work

In order to compare the model against other state-of-the-art models, we picked the model with the best performance so far, which was obtained after the fine-tuning (using the Adam optimizer, with a learning rate of 0.0005, freezing the first two modules and batch size 48), and adding a deformable convolution module along with a skip connection.

We compared the proposed model with other existing methods: U-Net, SegNet and Deeplabv3 models (models that we used as literature review for developing the architecture) and other recent architectures: WASP [35], PSANet [36], EMANet [37], AsymmetricNet [38], SANet [39], EfficientNet-L2 [40,41]. These models were chosen based on their number of parameters—to have around double of parameters than the proposed network.

The result of the proposed model on the three datasets can be seen in Table 18. We compared the performance on the Pascal VOC 2012 dataset, the Cityscapes dataset, and the ADE20K dataset.

The proposed model performs better than SegNet and U-Net on all three datasets, but weaker than the other models. All networks that have better results than the proposed one have a higher number of parameters (almost or more than double number of parameters).

The fact that it outperforms models with more number of parameters (SegNet and U-Net) shows that the model uses them better at learning. For PASCAL VOC 2012 dataset, EfficientNet-L2 has the highest performance, but the number of parameters is 18 times higher than the proposed method.

The difference in the number of parameters between the proposed model and the others is almost double, so the proposed model is capable of learning better and has a better overall performance. However, we want to keep the model lighter in terms of its size.

We compared the inference time with U-Net, SegNet and Deeplabv3 models (we considered only models that we used as literature review for developing the architecture). The inference time is better for the proposed model, as shown in Table 19.

## 5. Conclusions and Future Work

The paper presents a method for image segmentation using an encoder-decoder architecture based on the Xception classification model, that maintains a significant reduction in the number of parameters. The initial network went through a serious number of iterations: from increasing the resolution size and the depth to fine-tuning the network and experimenting with novel types of convolutions, dilated convolution, and deformable convolutions. We also employed the use of data augmentation.

We tested the network on three benchmark datasets, Pascal VOC 2012 (21 classes), Cityscapes (19 categories), and ADE20K (151 categories). The latter is very complex and difficult to segment. The proposed architecture reaches a 76.8 mean IoU on the Pascal VOC 2012 dataset and 58.1 on the Cityscapes dataset. It outperforms SegNet and U-Net, both networks having more parameters. Furthermore, the proposed model also has a better inference time.

As future work, we will increase the depth of the network along with changing and assessing the impact of the module of deformable convolutions, a new data augmentation method to help performance on smaller datasets, along with testing the performance on bigger datasets such as Microsoft COCO and Mapillary. Additionally, we will inspect the influence of data augmentation on the performance of the model by using GANs to generate realistic images for improving the performance of semantic segmentation networks.

## Figures and Tables

**Table 1:** Classes from the PASCAL VOC 2012 Dataset.

Group	Classes
person	person
animal	bird, cat, cow, dog, horse, sheep
vehicle	airplane, bicycle, boat, bus, car, motorbike, train
indoor	bottle, chair, dining table, potted plant, sofa, tv/monitor

**Table 2:** Classes from the Cityscapes Dataset.

Group	Classes
flat	road, sidewalk, parking, rail track
human	person, rider
vehicle	car, truck, bus, on rails, motorcycle, bicycle, caravan, trailer
construction	building, wall, fence, guard rail, bridge, tunnel
object	pole, pole group, traffic sign, traffic light
nature	vegetation, terrain
sky	sky

**Table 3:** Mean IoU for Pascal VOC 2012, Cityscapes and ADE20K datasets evaluated on U-Net [10], SegNet [11], DilatedNet [12] and DeepLabv3 [14].

	Mean IoU Pascal VOC 2012	Mean IoU Cityscapes	Mean IoU ADE20K
U-Net [10]	70.2	51.9	13.25
Seg-Net [11]	59.9	57	21.64
DilatedNet [12]	75.3	67.1	32.31
DeepLabv3 [14]	82.5	81	45.6

**Table 4:** Results of the architecture of the three datasets.

Dataset	Pixel Accuracy	Mean IoU
Pascal VOC2012	57%	20
Cityscapes	30%	16
ADE20K	30%	8

**Table 5:** Results of the architecture with transfer learning applied on the three datasets.

Dataset	Pixel Accuracy	Mean IoU
Pascal VOC 2012	73%	23
Cityscapes	82%	30
ADE20K	40%	12

**Table 6:** Results of the architecture with increased resolution size on the three datasets.

Model	Dataset	Pixel Accuracy	Mean IoU
	Pascal VOC 2012	72%	20
Simple	Cityscapes	74%	18
	ADE20K	41%	9
	Pascal VOC 2012	83%	44
With transfer learning	Cityscapes	80%	34
	ADE20K	52%	17

**Table 7:** Results of the architecture with transfer learning on Pascal VOC 2012 dataset (image resolution 299 × 299).

Variant (Resolution 299 × 299)	Pixel Accuracy	Mean IoU	Number of Parameters
ED model + 2 modules	87%	56	17M
ED model + 4 modules	89%	58	20M
ED model + 6 modules	85%	53	23M
ED model + 8 modules	75%	15	27M

**Table 8:** Results of the architecture with transfer learning on Pascal VOC 2012 dataset (image resolution 160 × 160).

Variant (Resolution 160 × 160)	Pixel Accuracy	Mean IoU
ED model + 2 modules	85%	44
ED model + 4 modules	87%	46
ED model + 6 modules	85%	44
ED model + 8 modules	75%	32

**Table 9:** Results on the datasets when using data augmentations.

Dataset	Pixel Accuracy	Mean IoU
Pascal VOC 2012	91%	60
Cityscapes	88%	45
ADE20K	70%	27

**Table 10:** The network’s performance when being trained with different optimizers (run three times, the values shown are the averages).

Optimizers	Dataset	Pixel Accuracy	Mean IoU
	Pascal VOC 2012	82.8%	46.06
SDG [26]	Cityscapes	84.8%	35.26
	ADE20K	66.1%	17.01
	Pascal VOC 2012	84.2%	52.98
RMSprop [27]	Cityscapes	86.9%	40.94
	ADE20K	71.7%	26.94
	Pascal VOC 2012	73.5%	24.94
Adadelta [28]	Cityscapes	76.9%	15.44
	ADE20K	55.0%	12.95
	Pascal VOC 2012	86.8%	60.33
Adagrad [29]	Cityscapes	87.6%	33.52
	ADE20K	72.3%	22.61
	Pascal VOC 2012	85.7%	59.36
Adam [30]	Cityscapes	88.3%	45.12
	ADE20K	71.3%	27.18
	Pascal VOC 2012	78.3%	39.41
Rectified Adam [31]	Cityscapes	87.0%	44.27
	ADE20K	71.1%	26.31

**Table 11:** Results after freezing the first n layers from the network. The best result is freezing after the fifth layer (which non-coincidently is after the second module of the network).

Frozen Layers	Datasets	Pixel Accuracy	Mean IoU
	Pascal VOC 2012	84.83%	60.79
1	Cityscapes	86.93%	41.49
	ADE20K	71.57%	27.11
	Pascal VOC 2012	85.34%	57.86
3	Cityscapes	86.37%	41.06
	ADE20K	71.32%	26.21
	Pascal VOC 2012	86.99%	58.72
5	Cityscapes	88.12%	45.11
	ADE20K	72.05%	28.12
	Pascal VOC 2012	86.44%	53.23
7	Cityscapes	86.67%	42.44
	ADE20K	70.65%	24.92
	Pascal VOC 2012	85.39%	55.85
9	Cityscapes	87.57%	41.27
	ADE20K	72.42%	27.67
	Pascal VOC2012	85.60%	51.28
11	Cityscapes	85.52%	41.68
	ADE20K	71.51%	25.53
	Pascal VOC 2012	84.21%	51.36
15	Cityscapes	84.96%	40.75
	ADE20K	72.11%-	26.65

**Table 12:** Results obtained by dilating convolution from each module from the entry flow in three different ways.

Method	Dataset	Pixel Accuracy	Mean IoU
	Pascal VOC 2012	89.51%	62.07
Atrous first convolution	Cityscapes	89.88%	47.32
	ADE20K	72.64%	27.69
	Pascal VOC 2012	85.73%	52.62
Atrous second convolution	Cityscapes	88.60%	44.14
	ADE20K	71.18%	24.00
	Pascal VOC 2012	88.26%	59.06
Both convolutions atrous	Cityscapes	87.27%	44.10
	ADE20K	30.71%	14.26

**Table 13:** Results obtained by dilating convolution from each module from the middle flow in four different ways.

Method	Dataset	Pixel Accuracy	Mean IoU
	Pascal VOC 2012	89.30%	62.64
Atrous first convolution	Cityscapes	88.71%	47.68
	ADE20K	72.91%	25.26
	Pascal VOC 2012	89.80%	65.01
Atrous second convolution	Cityscapes	89.15%	48.42
	ADE20K	73.94%	29.41
	Pascal VOC 2012	90.74%	66.77
Atrous third convolution	Cityscapes	89.53%	52.31
	ADE20K	74.22%	27.78
	Pascal VOC 2012	89.25%	63.61
All convolutions atrous	Cityscapes	88.65%	45.31
	ADE20K	72.93%	25.14

**Table 14:** Results obtained by adding an atrous spatial pyramid pooling (ASPP) module to the proposed model, between the middle flow and the upsampling flow.

Dilation Rates	Dataset	Pixel Accuracy	Mean IoU
	Pascal VOC 2012	90.78%	66.79
(3, 6, 9)	Cityscapes	90.28%	50.76
	ADE20K	76.51%	30.36
	Pascal VOC 2012	89.41%	62.22
(6, 12, 18)	Cityscapes	89.65%	50.89
	ADE20K	73.16%	25.67
	Pascal VOC2012	90.70%	67.94
(12, 24, 36)	Cityscapes	89.73%	51.37
	ADE20K	74.14%	27.48
	Pascal VOC 2012	90.63%	67.37
(24, 48, 72)	Cityscapes	89.33%	50.52
	ADE20K	73.16%	24.52

**Table 15:** Network results on the three datasets after employing the deformable convolutions.

Type	Dataset	Pixel Accuracy	Mean IoU
	Pascal VOC 2012	88.98%	66.58
Single deformable convolution	Cityscapes	81.64%	44.19
	ADE20K	72.75%	26.00
	Pascal VOC 2012	91.60%	73.57
A module of deformed convolution	Cityscapes	87.99%	55.58
	ADE20K	72.08%	28.94
	Pascal VOC 2012	90.40%	69.71
Three modules in series	Cityscapes	77.23%	50.45
	ADE20K	64.52%	16.91
	Pascal VOC 2012	91.46%	72.71
Three modules in parallel	Cityscapes	87.76%	52.18
	ADE20K	73.89%	25.00

**Table 16:** Performances of the best proposed model.

Dataset	Pixel Accuracy	Mean IoU
Pascal VOC 2012	94.92%	76.8
Cityscapes	92.82%	58.1
ADE20K	87.34%	35.7

**Table 17:** IoU per class for Pascal VOC 2012 and Cityscapes datasets.

Dataset	Classes										
Pascal Voc 2012	background	aeroplane	bicycle	bird	boat	bottle	bus	car	cat	chair	cow
	93.62	76.23	41.81	76.89	66.99	62.91	88.72	84.43	81.49	55.38	77.23
	dinning table	dog	horse	motorcycle	person	potted plant	sheep	sofa	train	TV	
	75.39	77.21	81.16	81.01	80.93	52.48	78.18	58.84	8.30	85.60	
Cityscapes	background	road	sidewalk	building	wall	fence	pole	traffic light	traffic sign	vegetation	terrain
	89.48	68.99	82.13	37.62	41.43	43.47	43.08	52.05	86.73	57.56	89.42
	sky	person	rider	truck	bus	train	motorcycle	bicycle			
	70.44	42.50	88.66	28.25	21.07	62.28	39.65	59.13			

**Table 18:** Results compared with other models.

Method	Pascal VOC 2012	Dataset (Mean IoU) Cityscapes	ADE20K	Number of Parameters
U-Net [10]	70.2	51.9	13.25	31M
SegNet [11]	59.9	57	21.64	30M
DeepLabv3 [14]	82.5	81	45.6	58M
WASP [35]	79.6	70.5	-	47M
PSANet [36]	85.7	80.1	43.7	102M
EMANet [37]	80.99	81.14	-	47M
AsymmetricNet [38]	-	81.3	45.2	63M
Eff-L2 [41]	90.5	-	-	485M
SANet [39]	83.2	-	-	55.5M
Proposed model	76.8	58.1	35.7	26M

**Table 19:** Inference time

Architecture	Inference Time
U-Net [10]	3 s
SegNet [11]	2.8 s
Deeplabv3 [14]	4.8 s
Proposed model	1.3 s
